# A *BRAF*-Negative Classic Hairy Cell Leukemia Patient with Long-Lasting Complete Remission after Rituximab and Pentostatin

**DOI:** 10.4274/tjh.galenos.2020.2020.0204

**Published:** 2020-11-19

**Authors:** Alessandro Gozzetti, Vincenzo Sammartano, Francesca Bacchiarri, Donatella Raspadori, Monica Bocchia

**Affiliations:** 1 University of Siena, Hematology, Siena, Italy; 2 Azienda Ospedaliera Universitaria, Siena, Italy

**Keywords:** Hairy cell leukemia, BRAF

## To the Editor,

The *BRAF *gene is mutated (V600E) in more than 95% of classic hairy cell leukemia (HCLc) cases [1,2,3], but cases have been reported of *BRAF *negativity in HCLc patients [4,5,6,7]. It has also been suggested to analyze mutations of exons 15 and 11 in the case of *BRAF *negativity [7]. Mutations in *MAP2K1* were identified in cases of the wild-type *BRAF* gene [8,9]. Limited data are available about these patients. We report here a patient with HCLc who had a long-lasting response to rituximab and pentostatin treatment. A 51-year-old woman was referred for lymphocytosis and fatigue. Physical examination revealed splenomegaly 5 cm below the costal margin. Laboratory findings confirmed lymphocytosis with white blood cell (WBC) count of 17.64x10^3^/µL, hemoglobin (Hb) of 11.4 g/dL, and platelet count of 187x10^3^/µL. A blood smear revealed 32% of cells with hairy features. Immunophenotyping of peripheral blood showed 45% of cells to be CD5-, CD19+, CD20+, CD11c+, FMC7+, CD25+, CD103+, CD123+, and lambda-restricted. The bone marrow aspirate was a dry tap and the bone marrow biopsy confirmed hairy cell infiltration of >90%, TRAP+, DBA44+/-, ANXA1+. A computed tomography (CT) scan confirmed splenomegaly. IGHV status was mutated and showed 96.88% homology with IGHV3-7*01 usage. Mutation analysis of *TP53* performed by polymerase chain reaction and DNA direct sequencing of exons 2 through 10 revealed a wild-type status. Allele-specific PCR for *BRAF * V600E, T599I, V600M, and K601E at exon 15 and G464E, G464V, G466R, G466A, G466V, G466E, G469R, G469A, G469V, G469E, and V471F at exon 11 did not detect mutations. PCR and direct DNA Sanger sequencing of both exons 15 and 11 did not reveal mutations. A diagnosis of *BRAF*-negative HCLc was made. Due to the presence of fatigue in a relatively young woman with disease-related anemia, she was treated with cladribine (CD) at a total dose of 10 mg daily for 5 days subcutaneously, but splenomegaly was still present 2 cm below the costal margin 4 months later, and hairy cells were still present at a rate of 50% in bone marrow biopsy. After 10 months she developed severe neutropenia (WBC count 2.1x10^3^/µL, neutrophils 4%, hairy cells 25%, Hb 10.5 g/dL, platelets 142x10^3^/µL). Rituximab at 375 mg/m^2^ IV and pentostatin at 4 mg/m^2^ every 14 days were administered a total of eight times. Normalization of blood counts and absence of hairy cells was observed by bone marrow biopsy and flow cytometry 4 months later. The CT scan showed normal spleen diameters. At the last follow-up (78 months after therapy), the bone marrow aspirate and biopsy still confirmed complete recovery. Hematologic values were normal: WBC count 5.1x10^3^/µL, neutrophils 56%, Hb 13.5 g/dL, and platelets 182x10^3^/µL. Splenic diffuse non-Hodgkin lymphoma could be excluded by the presence of TRAP+, ANXA1+, and CD123+ cells. Few *BRAF*-negative HCLc patients have been reported (Table 1), with 11/53 pretreated patients with HCLc in one study, without data related to response [4]. Another study reported 2 patients being *BRAF*-negative at exon 15, with one responsive to CD and the other to splenectomy [5]. One study reported 1 patient negative at exon 15 and responsive to CD [6]; another reported 3 patients negative at exon 15, two of whom showed a mutation at exon 11 [7], and another patient responsive to CD was reported [10]. More cases need to be studied.

## Figures and Tables

**Table 1:**
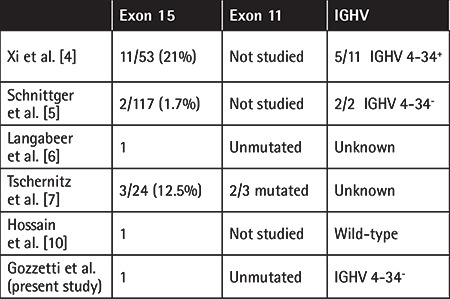
*BRAF* wild-type HCLc cases reported in the current literature.
